# Long Non-coding RNA *H19* Regulates Porcine Satellite Cell Differentiation Through *miR-140-5p*/*SOX4* and *DBN1*

**DOI:** 10.3389/fcell.2020.518724

**Published:** 2020-11-25

**Authors:** Jingxuan Li, Tao Su, Cheng Zou, Wenzhe Luo, Gaoli Shi, Lin Chen, Chengchi Fang, Changchun Li

**Affiliations:** ^1^Key Laboratory of Agricultural Animal Genetics, Breeding and Reproduction of the Ministry of Education, Huazhong Agricultural University, Wuhan, China; ^2^Key Laboratory of Swine Genetics and Breeding of the Ministry of Agriculture, Huazhong Agricultural University, Wuhan, China; ^3^Shandong Provincial Key Laboratory of Animal Disease Control and Breeding, Institute of Animal Science and Veterinary Medicine, Shandong Academy of Agricultural Sciences, Jinan, China; ^4^The Cooperative Innovation Center for Sustainable Pig Production of Hubei Province, Wuhan, China

**Keywords:** *H19*, porcine satellite cells, differentiation, *miR-140-5p*, *SOX4*, *DBN1*

## Abstract

The *H19* gene promotes skeletal muscle differentiation in mice, but the regulatory models and mechanisms of myogenesis regulated by *H19* are largely unknown in pigs. Therefore, the regulatory modes of *H19* in the differentiation of porcine skeletal muscle satellite cells (PSCs) need to be determined. We observed that *H19* gene silencing could decrease the expressions of the myogenin (*MYOG*) gene, myogenic differentiation (*MYOD*), and myosin heavy chain (*MYHC*) in PSCs. Therefore, we constructed and sequenced 12 cDNA libraries of PSCs after knockdown of *H19* at two differentiation time points to analyze the transcriptome differences. A total of 11,419 differentially expressed genes (DEGs) were identified. Among these DEGs, we found through bioinformatics analysis and protein interaction experiment that SRY-box transcription factor 4 (*SOX4*) and Drebrin 1 (*DBN1*) were the key genes in *H19*-regulated PSC differentiation. Functional analysis shows that *SOX4* and *DBN1* promote PSC differentiation. Mechanistically, *H19* regulates PSC differentiation through two different pathways. On the one hand, *H19* functions as a molecular sponge of *miR-140-5p*, which inhibits the differentiation of PSCs, thereby modulating the derepression of *SOX4*. On the other hand, *H19* regulates PSC differentiation through directly binding with DBN1. Furthermore, *MYOD* binds to the promoters of *H19* and *DBN1*. The knockdown of *MYOD* inhibits the expression of *H19* and *DBN1*. We determined the function of *H19* and provided a molecular model to elucidate *H19*’s role in regulating PSC differentiation.

## Introduction

Myogenesis in the adult pig is a highly regulated process that includes the activation of muscle stem cells, proliferation and differentiation of myoblasts, and cell fusion to form multinucleated myotubes, which eventually differentiate into muscle fiber (Li et al., 2016, 2019; Zhu et al., 2017). The canonical adult myogenic progenitors, named satellite cells (SCs) and located between the basement membrane and the muscle sarcolemma in myofibers, are essential for skeletal muscle maintenance, regeneration, and differentiation (Wang and Rudnicki, 2011; Relaix and Zammit, 2012; Ding et al., 2017; Zammit, 2017). Several long non-coding RNAs (lncRNAs) are involved in this process. For example, lncRNA *AK017368* can promote proliferation and restrain differentiation of C2C12 cells (Liang et al., 2018). lncMD can promote the differentiation of bovine myoblasts by acting as a ceRNA to sequester *miR-125b*, thereby leading to the rise of the insulin-like growth factor 2 (*IGF2*) expression level (Sun et al., 2016). Recently, Jin et al. (2018) found that the *SYISL* gene recruits the enhancer of zeste homolog 2 (EZH2) protein to the promoters of the cell-cycle inhibitor gene *p21* and muscle-specific genes and further regulates proliferation and differentiation of SCs in mice.

*H19* is among the best-known lncRNAs and also has a critical function in skeletal muscle differentiation (Giovarelli et al., 2014; Liu et al., 2017). For example, *H19*-encoded microRNAs miR-675-5p and miR-675-3p can execute the prodifferentiation function of *H19* in skeletal muscle lacking endogenous *H19* (Dey et al., 2014). Xu et al. (2017) also demonstrated that *H19* could promote the differentiation of bovine skeletal muscle SCs by suppressing Sirt1/FoxO1. These discoveries demonstrate that *H19* plays an important role in porcine skeletal muscle SC (PSC) differentiation and myogenesis through different molecular mechanisms in different species or different tissues. However, the function of *H19* gene in PSC differentiation is poorly understood. Meanwhile, we aimed to explore new molecular mechanisms in this process.

In this study, we found that *H19* is essential and required for PSC differentiation. We performed transcriptome analysis of PSCs after knockdown of *H19* to identify the candidate genes regulated by *H19* and their potential roles in PSC differentiation. The candidate target genes of *H19*, microRNA *miR-140-5p* and mRNA SRY-box transcription factor 4 (*SOX4*), were predicted by bioinformatics analysis and validated *in vitro*. Simultaneously, the function of *miR-140-5p* and *SOX4* in PSC differentiation was explored. Moreover, we used a pull-down experiment to find the protein binding with *H19*. Then, we explored the role of this protein in cell differentiation. In summary, this study aimed to facilitate the understanding of the mechanisms of *H19* in PSC differentiation and construct a potential gene network between *H19* and the target genes affecting this process.

## Materials and Methods

### Animals and PSCs Isolation

Animal care and all the experimentation in this study were carried out in accordance with the preapproved guidelines from Regulation Proclamation No. 5 of the Standing Committee of Hubei People’s Congress. All experimental protocols were approved by the Ethics Committee of Huazhong Agricultural University, Wuhan City, Hubei Province, China.

Seven tissues, including myocardium, lung, stomach, skeletal muscle (the skeletal muscle tissue was collected from the biceps femoris), lymph, kidney, and encephalon, were collected strictly according to the anatomy of the pigs. All tissue samples came from three 2-year-old Yorkshire boars.

All the PSCs were isolated from 3-day-old Yorkshire male pigs. The muscle of extremities (including triceps, biceps femoris, semitendinosus, semimembranosus, and gastrocnemius) were collected and kept in PBS supplement with 1% antibiotic-antimycoti. The tissues were dissected and digested with collagenase II (Gibco, Life Technologies, United States, Cat#17101-015, 1 mg/mL) in a 37°C water bath shaking table for 2 h. After digestion was termination with DMEM, including 10% FBS. The mixture was filtered through a 100-μm cell strainer and centrifuged at 2500 r/min for 15 min. Then, we removed the supernatant, and the precipitation was resuspended by PBS and centrifuged at 2200 r/min for 12 min. Next, 70- and 40-μm cell strainers were continued and the supernatant similarly removed and then resuspended with RPMI 1640 (Gibco, United States, Cat#A10491-01) and centrifuged at 2000 r/min for 10 min. Last, the cells were suspended with 20% FBS (Gibco, Life Technologies, United States, Cat#10270) and transferred to the culture dish. After 2.5 h, the cell suspension was transferred to the new culture dishes, which were coated with Matrigel (Corning, BD, United States, Cat# 356234); the cells left in the original cell culture dishes are fibroblasts.

### PSCs Culture and Differentiation

Isolated PSCs were cultured on Matrigel (BD, Cat# 356234) coated 10-cm plates (Corning Cat#430167) in PM + medium (PM + medium containing 76.5% RPMI 1640 (Life, Cat#A10491), 20% FBS, 0.5% chicken embryo extract (GEMINI, Cat#100-163P), 1% GlutaMax (Gibco, Life Technologies, United States, Cat#35050-061), 1% non-essential amino acids (Gibco, Life Technologies, United States, Cat#11140-050), 1% antibiotic-antimycotic (Gibco, Life Technologies, United States, Cat#15240-062), and 2.5 ng/mL human recombinant basic fibroblast growth factor (Gibco, Invitrogen, United States, Cat#13256029). When cells grow to 70%, they can be inoculated into six-hole plates or new culture dishes for subsequent experiments. In order to separate SCs from fibroblast cells, the cells were incubated in an uncoated dish at 37°C for 2.5 h and then transferred to a new Matrigel-coated dish.

To observe the formation of myotubes, PSCs were differentiated in DMEM containing 5% horse serum (HyClone, Cat#SH30074.02) for different time points, changing the culture medium every day.

### 5′ and 3′ Rapid Amplification of cDNA Ends (RACE) and Full-Length lncRNA Cloning

To determine the transcription start points and the full length of the *H19* transcripts, the primary myoblasts were collected for total RNA extraction by using RNAiso reagent (Takara, Japan, 9109). There were approximately 2 × 10^6^ cells for the experiment. Then, the 5′ and 3′ rapid amplification of cDNA ends (RACE) experiments were carried out. We used the Takara SMARTer RACE cDNA Amplification Kit (Clontech, United States, 634858) according to the manufacturer’s instructions, and the gene-specifc primers used for PCR were as follows:

GSP1 (ACTCGCTTGAGATGCTCTTTCCACCTG)
GSP2-1 (GGTCAATTTTGGTTTCAGGTCGTGGC)
GSP2-2 (CCAGGTGGAAAGAGCATCTCAAGCG)

The PCR products were separated on 1.5% agarose gels, and the bands were extracted and inserted into the pRACE vector. The clones were selected randomly to perform PCR amplification. Then, the positive clones were sent to the company for Sanger sequencing. Sequences were aligned with BLAST in the NCBI standard nucleotide BLAST.

### Nuclear and Cytoplasmic RNA Fractionation

We collected PSCs in proliferative and differentiated periods, respectively; they were washed twice with cold PBS and centrifuged at 500 *g* for 3 min. The 0.2 mL lysis buffer [50 mM Tris-HCl pH 8.0, 140 mM NaCl, 1.5 mM MgCl_2_, 0.5% Octylphenoxy poly (ethyleneoxy) ethanol IGEPAL CA-630, 1 U/μL RNase inhibitor, 1 mM DTT] was used to resuspend the lipid precipitation, which was then placed on ice for 5 min and centrifuged at 500 *g* for 3 min at 4°C. The supernatant was transferred to a new 1.5-mL microcentrifuge tube without RNase and centrifuged at 14,000 r/min for 1 min. The supernatant was transferred to another microcentrifuge tube, 1 ml Trizol was added, and they were mixed, which is the cytoplasmic RNA. The nuclear RNA was mixed in the precipitation. The deposit was washed with 0.2 mL lysis buffer, and all nuclear and cytoplasmic RNA were extracted with 1 mL TRIzol.

### Transfection of siRNAs

We used Lipofectamine 2000 (Invitrogen, Life Technologies, United States, Cat#11668019) to transfect siRNA or ASO specific for *H19*, *SOX4*, Drebrin 1 (*DBN1*), and *MYOD*. The siRNA sequences were as follows:

si*H19*-1
(CCUAUAGGCAGGGCAACAUTTdT; AUGUUGCCCUGCCUAUAGGTTdT)
si*H19*-2
(GAGCACACAUGGGUACCUUTTdT; AAGGUACCCAUGUGUGCUCTTdT)
si*H19*-3
(CCUCCUAGCUCUGACUCAATTdT; UUGAGUCAGAGCUAGGAGGTTdT)
*SOX4* ASO (GTACTTGTAGTCGGGGTAGT)
*DBN1* siRNA
(GGAGUUUGCCCAAUCGGAATTdT; UUCCGAUUGGGCAAACUCCTTdT)
*MYOD* siRNA
(GCUACGACGGCACCUAUUATTdT; AAUAGGUGCCGUCGUAGCTTdT)
Negative control (NC) siRNA
(UUCUCCGAACGUGUCACGUTTdT; ACGUGACACGUUCGGAGAATTdT).

The *miR-140-5p* mimic, inhibitor, mimic NC, and inhibitor NC were as follows:

*miR-140-5p* mimic
(AGUGGUUUUACCCUAUGGUAGdT; ACCAUAGGGUAAAACCACUUUdT)
*miR-140-5p* inhibitor (CUACCAUAGGGUAAAACCACU)
mimic NC and inhibitor NC (CAGUACUUUUGUGUAGUACAA)

The RNA oligo against porcine *H19*, *miR-140-5p*, *DBN1*, and *MYOD* were purchased from genepharma. The ASO against *SOX4* were purchased from Sangon Biotech (Shanghai). The 50 nM RNA oligo or ASO specific to each gene (and NC siRNA) were transfected to PSCs in PM + medium and transferred to DM after 24 h. Then, the cells were harvested on DM 36 h.

### Plasmid Construction and Cell Transfection

For the *H19*, *SOX4*, and *DBN1* overexpression plasmids, full-length sequences were cloned into the pcDNA3.1 plasmid. The truncated *H19* were obtained by PCR using H19-pcDNA3.1 plasmid as a template and then were cloned into pcDNA3.1. Full-length *H19*, *H19*-mutant, *SOX4*, and *DBN1* sequences were amplified according to primers (Supplementary Table S2). For cell transfection, PSCs were transfected with 3 μg of the expression vectors in each well of a six-well plate. Then, the cells were harvested on DM 36 h.

### Isolation of Total RNA and Performance of Quantitative Polymerase Chain Reaction (qPCR)

Total RNA was extracted using the RNAiso reagent (Takara, Japan, 9109) from the PSCs following the manufacturer’s instructions. The RNA samples were quantitated and subjected to quality inspection. cDNA synthesis for mRNA detection was carried out using the RevertAid First Strand cDNA Synthesis Kit (Thermo, China, Cat#k1622). Quantitative polymerase chain reaction (qPCR) for mRNA detection was carried out in the Roche LightCyler 480 system (Roche, Mannheinm, Germany) using SYBR Green (CWBIO, China, CW0957) according to the manufacturer’s instructions. The quantitative real-time polymerase chain reaction (qRT-PCR) data were analyzed using the 2^–ΔΔCT^ method as previously described (Anders et al., 2015). The relative fold changes of mRNA expression were quantified by normalizing the cycle threshold (CT) value of the experimental gene to the mean CT value of the control *18s* gene.

### Western Blotting and Antibodies

The protein expression levels of the myogenin (MYOG) gene, myogenic differentiation (MYOD), and myosin heavy chain (MYHC), DBN1, SOX4 in PSCs were detected by performing immunoblotting. Transfected cells were lyzed in RIPA buffer with 1% PMSF and loaded protein onto a SDS-PAGE gel and transferred them onto a PVDF membrane, and non-specific binding was blocked with 5% non-fat milk in Tris-buffered saline with Tween 20 for 4 h. Then, they were incubated with 1:500 diluted polyclonal mouse MYOG (Abcam, United Kingdom, Cat#ab1835), 1:1000 diluted polyclonal rabbit MYOD (Abclonal, China, Cat#A0671), 1:3000 diluted polyclonal mouse MYHC antibody (Milipore, China, Cat#05-716), 1:1000 diluted polyclonal rabbit DBN1 (Abclonal, China, Cat#A6366), and 1:1000 diluted polyclonal rabbit SOX4 (Bioss, China, Cat#bs-11208R) at 4°C overnight, respectively. The blots were subsequently incubated with HRP conjugated secondary antibody (1:4000), including HRP-labeled goat antimouse IgG (Servicebio, China, Cat#GB23301) and HRP-labeled goat antirabbit IgG (Servicebio, China, Cat#GB23303). ECL substrates were used to visualize signals (Beyotime, China, Cat#P0018A). β-Tubulin (Servicebio, China, Cat#GB13017-2) was used as an endogenous protein for normalization. Image J software was used to conduct quantitative analysis of Western blotting results according to the gray value of the strip.

### Immunofluorescent Analysis of Cultured Cells

Cultured SCs were fixed with ice-cold 4% paraformaldehyde (in PBS) for 15 min, rinsed with PBS, and permeabilized in 0.3% Triton X-100 (in PBS) for 10 min. Fixed and permeabilized cells were blocked with blocking solution [3% bovine serum albumin (BSA), 0.3% TritonX-100, 10% FBS complemented with PBS] and incubated with 1:1000 diluted polyclonal mouse MYHC (Milipore, China, Cat#05-716) antibodies overnight at 4°C. Then, washing cells with PBS, the cells were incubated with Alexa 594-labeled donkey antimouse IgG antibodies (Antgene, China, Cat#ANT029) for 1 h at room temperature. Last, the cells were stained with Hoechst 33342 (Sanofi-Aventis, Germany, C1022) for 10 min and washed with PBS twice. All images were acquired by a Leica SP8 confocal microscope and processed with Adobe Photoshop CS6 to adjust brightness and contrast for publication. Exposure, contrast, brightness, and other image parameters were the same in both the experimental and control groups. Immunofluorescence results were quantified by Image J software.

### Library Construction and Sequencing

In total, 3 μg of RNA for each sample was used to construct sequencing libraries. The libraries were sequenced on an Illumina Hiseq^TM^ 2500 platform, and 100 bp paired-end reads were generated. Next, we use the HISAT2 (version 2.0.2) to get the qualified and clean reads mapped to the pig reference genome (Sus scrofa 11.1), and stringTie (version 1.2.2) was used to assemble the mapped reads with default parameters.

### Identification of Differentially Expressed Genes (DEGs)

First, the expression level of each gene was calculated using the HTseq software (0.6.1) and normalized based on the reads per FPKM method (Mortazavi et al., 2008; Anders et al., 2015). Subsequently, differentially expressed genes (DEGs) were identified using the R packages DEGseq 2 (Wang et al., 2010).

### Cluster Analysis and Functional Annotation of DEGs

Cluster analysis was conducted using the Pheatmap software in R package to identify genes with similar expression patterns, which often have similar functions. GO and KEGG pathway enrichment analyses were performed by DAVID analysis by running queries for each protein-coding gene against the DAVID database.

### RNA Fluorescent *in situ* Hybridization (FISH)

Cells were briefly rinsed in PBS and fixed in 4% formaldehyde in PBS for 10 min at room temperature. Then, the cells were permeabilized in PBS containing 0.5% Triton X-100. The experiments were performed using a fluorescent *in situ* hybridization (FISH) Kit (Guangzhou RiboBio Co. Ltd., Guangzhou, China) according to the manufacturer’s instructions in the following steps. Briefly, the cells were incubated with the RNA probes in hybridization buffer overnight at 37°C. The cells were washed three times with different concentrations of SSC buffer, stained with 4′, 6-diamidino-2-phenylindole (DAPI) for 10 min at room temperature, and then rinsed in PBS and, finally, examined using a confocal laser-scanning microscope.

### Luciferase Reporter Assay

Wild-type (WT) *H19*, mutated *H19*, WT *SOX4*, or mutated *SOX4* sequence were inserted into pGL3-basic vector. The reconstructed plasmids were transfected into PSCs. PSCs were cultured in 24-well plates, the cells were harvested after 24 h of transfection, and luciferase assays were performed with the Dual-Luciferase Reporter Assay System (Promega, Madison, WI, United States).

### Pull-Down Assay With Biotinylated miR-140-5p (Biotin-miR-140-5p)

A biotin pull-down assay was performed as previously described (Yu et al., 2017; Zhu et al., 2017). After 48 h of PSCs transfected with biotin-*miR-140-5p* or biotin-*miR-140-5p*-mut, the cells were washed with PBS, followed by incubation in a lysis (10 mM KCl, 1.5 mM MgCl2, 10 mM Tris-HCl at pH 7.5, 5 mM dithiothreitol) with RNase inhibitor (Thermo Fisher) and proteinase inhibitor cocktail (Roche) buffer for 30 min. The 500 μL NaCl (1 M) and 50 μl beads (Invitrogen) were added into the supernatant after it was centrifuged for 5 min at 12,000 *g*. The beads were blocked for 12 h in lysis buffer, including BSA, before use. Then the lysates were incubated with beads at 4°C for 4 h. After incubation, the beads were washed five times using washing buffer (5 mM Tris-HCl pH 7.5, 0.5 mM EDTA, 1 M NaCl). Trizol was used to extract RNA and evaluated by qPCR assay.

### RNA Pull-Down Assay

RNA pull down was performed as previously described. In brief, full-length sense and antisense *H19* were amplified by PCR and cloned using a pcDNA3.1 Vector. Biotin-labeled RNAs were generated by an *in vitro* transcription reaction with Biotin RNA Labeling Mix and T7 RNA polymerase (Roche Life Science) and then treated with Dnase I and EDTA. In total, 3 μg of biotinylated RNA was incubated with proteins extracted from PSCs and then the RNAs were targeted with streptavidin beads. Finally, the protein complexes associated with the beads were analyzed by mass spectrometry (MS) and western blot.

### RNA Immunoprecipitation (RIP) Assay

For RNA immunoprecipitation (RIP), cells were lyzed with cell lysis buffer (Cell Signaling Technology) supplemented with PMSF (Genstar). RIP was performed using the Magna RIP RNA-Binding Protein Immunoprecipitation Kit (Millipore, Burlington, MA, United States) according to the manufacturer’s instructions. The antibody against DBN1 was used for RIP. The co-precipitated RNAs were detected by reverse-transcription polymerase chain reaction assay.

### Colocalization of lncRNA and Protein

For colocalization analysis of *H19* and DBN1, cells were first hybridized with anti-*H19* probe. Then the cells were rinsed in PBS and incubated with 1:100 diluted polyclonal rabbit DBN1 antibody (Abclonal, China, Cat# A6366) at 4°C overnight. The next day, the cells were incubated in the specified secondary antibodies and counterstained with DAPI. Images were obtained with a confocal laser-scanning microscope.

### Chromatin Immunoprecipitation (ChIP) Assays

Chromatin immunoprecipitation (ChIP) assay was performed using the ChIP Kit (Beyotime, P2078) according to the manufacturer’s instructions. Each ChIP reaction was performed using 10^7^ cells and 10 μg of antibodies against MYOD (Abclonal, China, Cat#A0671). IgG was used as the negative control. Fold enrichment was quantified using qPCR.

### Statistical Analysis

All results were presented as mean ± standard error of mean (SEM). Multigroup comparisons of the means were carried out by a one-way analysis of variance test with *post hoc* contrasts by the Student–Newman–Keuls test. The two-tailed Student’s *t-*test was performed for significance analysis by using SAS software. *P* < 0.05 indicated the significant difference.

## Results

### Characterization of *H19* and Identification of Its Function in PSCs

**FIGURE 1:**
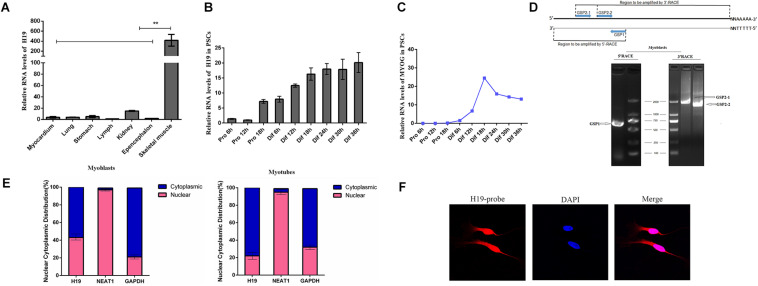
Expression pattern and characterization of H19. **(A)** Real-time PCR analysis of H19 expression in different tissues of Yorkshire. Mean values ± SEM, *n* = 3, **P < 0.01. **(B)** Real-time PCR analysis of H19 expression in PSCs during the period of proliferation and differentiation. There is a 6-h interval between each period. Mean values ± SEM, *n* = 3. **(C)** Real-time PCR analysis of MYOG, MYOD, and MYHC expression in PSCs during the period of proliferation and differentiation. There is a 6-h interval between each period. Mean values ± SEM, *n* = 3. **(D)** Agarose gelelectrophoresis showing the 5′ and 3′ RACE results of H19 for 2 days’ proliferation. Left, 5′RACE product of GSP1 (710 bp); right, 3′RACE product of GSP2-1 (1922 bp) and GSP2-2 (1597 bp). **(E)** RNA of PSCs was fractionated into nuclei and cytosol fractions. H19 subcellular distribution was calculated by qPCR assay data, the graphs showed the expression in myoblasts and myotubes, and NEAT1 and GAPDH were used as the nuclear control and cytoplasmic control, respectively. **(F)** Confocal FISH images showing localization of H19 in PSCs.

We detected the expression pattern of *H19* in different tissues using qRT-PCR to explore the function of *H19* in porcine myogenesis. As shown in Figure 1A, *H19* features a relatively high expression level in skeletal muscle tissues. The expression pattern of *H19* in PSCs was observed at different proliferation and differentiation time points. H19 had a higher expression level in the differentiation period than in the proliferation period, and its expression level increased with differentiation time points (Figure 1B), thereby suggesting the possible role of *H19* in PSC differentiation. We also detected the expression level of three marker genes in PSCs (Figure 1C and Supplementary Figure S1). The full-length cDNA sequence of *H19* was identified by performing 5′ and 3′ RACE. *H19* is a 2280-nucleotide transcript with a poly (A) tail (Figure 1D). The full-length sequence of *H19* is shown in Supplementary Sequence S1. The conservation of *H19* was analyzed in human, mouse, and pig (Supplementary Figure S2), and it showed that *H19* was partially conservative. Furthermore, nuclei and cytosol RNA were partitioned from PSCs to detect the cellular location of *H19* by qRT-PCR analysis. *H19* transcript was located in nearly equal amounts in the cytoplasm and nucleus at the proliferation stage (myoblasts). However, when cells differentiate (myotubes), higher *H19* expression was observed in the cytoplasm than in the nucleus (Figure 1E). Simultaneously, the results from FISH also showed that *H19* was located in both the nucleus and cytoplasm of PSCs (Figure 1F and Supplementary Figure S3).

**FIGURE 2:**
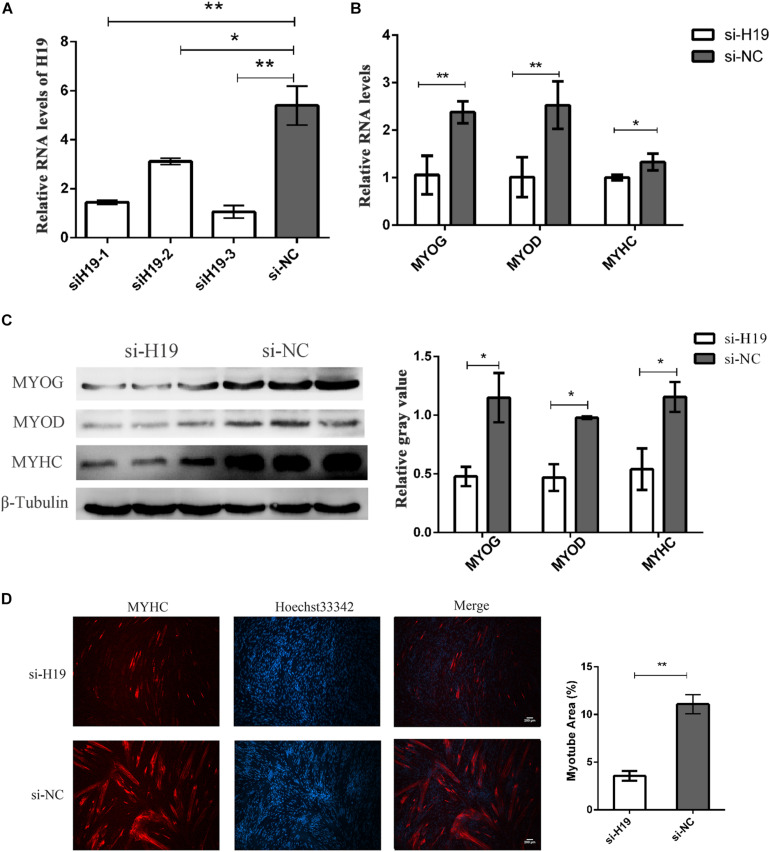
The knockdown of H19. **(A)** Screening assay of siRNA of H19 showed the third siRNA that have the highest interference efficiency. **(B)** Knockdown of H19 decreased the mRNA levels of MYOG, MYOD, and MYHC. **(C)** Knockdown of H19 decreased the levels of MYOG, MYOD, and MYHC protein. The gray level of WB was calculated by Image J software. Each experiment had three biological replicates. **(D)** Knockdown of H19 inhibited the differentiation of PSCs. PSCs transfected with either H19 siRNA or NC siRNAs were induced to differentiate. After differentiation for 24 h, cells were fixed and stained for MYHC, the marker of muscle differentiation. The fluorescence intensity and myotube area were calculated by Image J software. Each experiment had three biological replicates. Mean values ± SEM, *n* = 3. *p < 0.05, **p < 0.01.

We knocked down *H19* in PSCs to further determine the role of *H19* in skeletal muscle cell differentiation. Three independent small interfering RNA (siRNA) specific to porcine *H19* were designed and transfected in PSC cultured in growth and differentiation mediums after 12 h proliferation with NC siRNA as NC. The cells were harvested at 24 and 36 h after induced differentiation. si*H19*-3 had the highest knockdown efficiency as shown in Figure 2A. Hence, si*H19*-3 was used for subsequent experiments. Successful knockdown of *H19* significantly inhibited PSC differentiation as proven by the reduced expression of *MYOG*, *MYOD*, and *MYHC* and the decreased number of positive myotubes (Figures 2B–D). *H19* may promote PSC differentiation into myotubes.

### Transcriptome Data Analysis After Knockdown of *H19* in PSCs

**FIGURE 3:**
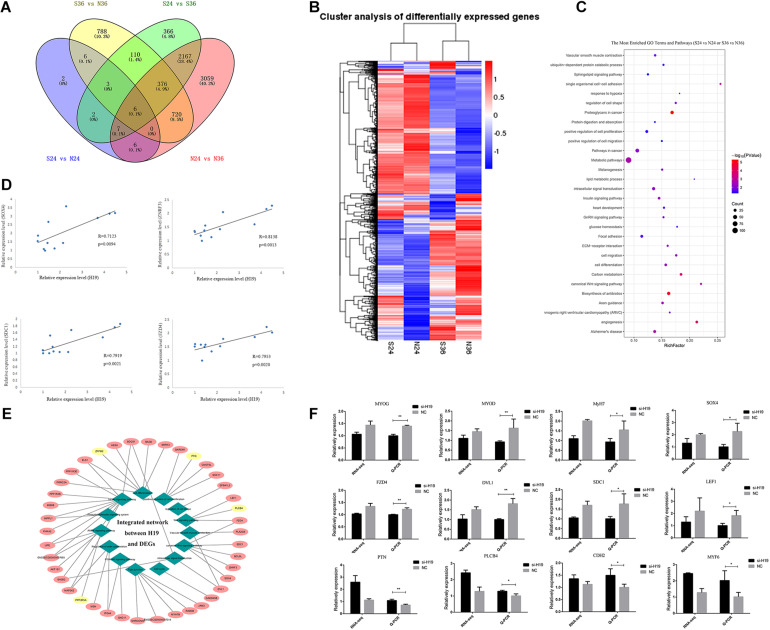
The analyses of DEGs. **(A)** Venn diagrams of the DEGs in different comparisons. **(B)** Hierarchical clustering dendrogram analyses were conducted with 7617 overlapped DEGs among four different comparative groups (S24 versus N24, S36 versus N36, N24 versus N36, S24 versus S36). Each column represents a sample, and each row represents a DEG. The colors correspond to a normalized expression value of each transcript (red: high relative expression; blue: low relative expression). **(C)** The most enriched top 30 GO terms and KEGG pathways. Columns from bottom to top represent *P*-values from small to large. **(D)** Linear regression of H19 and selected DEG expression. The R and P indicate the Pearson correlation coefficient and *p* value of each pair of H19 and DEGs in 12 samples (three for si24, three for NC24, three for si36, three for NC36). **(E)** Integrated network analysis of H19 and selected DEGs. Green rhombus represents different GO terms and pathways, similar processes are classified into one category, and 14 major categories exist. Pink and yellow ellipses represent positive and negative regulation between H19 and DEGs, respectively. **(F)** Verification of sequencing data by Q-PCR. Comparisons of relative expression levels for 12 DEGs. Comparisons of relative FPKM in the left; comparisons of relative expression levels in the right. Mean values ± SEM, *n* = 3. *p < 0.05, **p < 0.01.

We constructed 12 cDNA libraries (including si24h, NC24h, si36h, and NC36h groups; each group observation was repeated thrice) from two differentiation time points (24 and 36 h) after knockdown of *H19* and sequenced them on an Illumina HiSeq^TM^ platform to find the network pathways regulated by *H19* and systematically identify transcriptome change after knockdown of *H19*. After sequencing, 11,419 DEGs were detected between different groups, including si24 versus NC24, si36 versus NC36, NC24 versus NC36, and si24 versus si36, which yielded 32, 2009, 6341, and 3037 differentially expressed mRNAs, respectively (Figure 3A). We performed hierarchical cluster analysis based on expression abundance to gain insights into the expression patterns of DEGs in the libraries. As shown in Figure 3B, si24-NC24 and si36-NC36 clustered into individual groups, in which the DEGs exhibited distinguishable expression patterns.

We performed GO and KEGG pathway enrichment analyses and obtained 185 significantly enriched GO terms in the biological process and 140 KEGG pathways to elucidate further the functional roles of DEGs in skeletal muscle SC differentiation (Figure 3C and Supplementary Figure S4A). Among the GO terms and KEGG pathways, cell differentiation, positive regulation of cell proliferation, cell proliferation, insulin signaling pathway (Di Camillo et al., 2014), PI3K-Akt signaling pathway (Xiao et al., 2014; Yao et al., 2014; Li et al., 2018; Ueda-Wakagi et al., 2018), and Wnt signaling pathway (Otto et al., 2008; Rudnicki and Williams, 2015; Girardi and Le Grand, 2018) are related to skeletal muscle development, indicating that *H19* may play a certain role in myogenesis.

The relationship between *H19* and other DEGs was further investigated by analyzing the expression regulation of *H19* on selected DEGs enriched in muscle-related pathways in four groups (si24 versus NC24, si36 versus NC36, NC24 versus NC36, si24 versus si36) according to the relative expression levels at *p* < 0.05 (Figure 3D and Supplementary Figure S4B). A total of 41 DEGs were selected, among which 37 exhibited a positive relationship with *H19*, and four presented negative relevance with *H19*. Among these 41 genes, the genes involved in the Wnt signaling pathway, such as Zinc and ring finger 3 (*ZNRF3*), *SOX4*, syndecan-1 (*SDC1*), and frizzled class receptor 4 (*FZD4*), exhibited a higher correlation coefficient with *H19*, thereby suggesting the possible interaction between *H19* and Wnt signaling pathways. The integrated network is shown in Figure 3E. qRT-PCR was used to validate DEGs in si36 and NC36, and the qRT-PCR results were consistent with the sequencing data (Figure 3F). Therefore, Wnt signaling pathways were identified as the key factors responsible for the differentiation of PSCs.

### *SOX4* Is Required for PSC Differentiation

**FIGURE 4:**
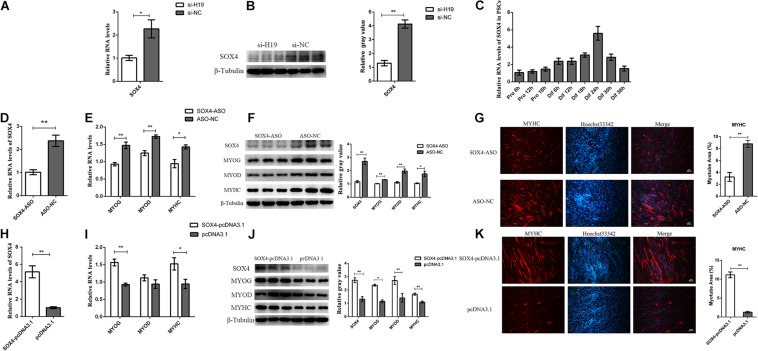
Functional research of the SOX4 gene. **(A)** Real-time PCR analysis of SOX4 after the knockdown of H19. **(B)** The protein level of SOX4 after the knockdown of H19. **(C)** Real-time PCR analysis of SOX4 expression in PSCs during the period of proliferation and differentiation. There is a 6-h interval between each period. **(D)** The efficiency of SOX4 ASO. **(E)** Interference of SOX4 decreased the mRNA levels of MYOG, MYOD, and MYHC. **(F)** Knockdown of SOX4 decreased the protein levels of MYOG, MYOD, and MYHC. The gray level of WB was calculated by Image J software. **(G)** Representative photographs of MYHC immunofluorescence staining in PSCs differentiated for 24 h showing that SOX4 knockdown significantly decreased the MYHC protein expression level. The myotube area was calculated by Image J software. Each experiment had three biological replicates. **(H)** Real-time PCR analysis of SOX4 expression in PSCs transfected with control vector or SOX4-pcDNA3.1 vector. **(I)** Overexpression of SOX4 increased the mRNA levels of MYOG and MYHC but did not affect MYOD expression. **(J)** Overexpression of SOX4 significantly promoted the protein levels of MYOG, MYOD, and MYHC. **(K)** Representative photographs of MYHC immunofluorescence staining in PSCs differentiated for 24 h showing that SOX4 overexpression significantly increased the MYHC protein expression level. **(G)** and **(K)** were two separate experiments, and the cell seeding densities were different. Mean values ± SEM, *n* = 3. *p < 0.05, **p < 0.01.

Among the genes in the Wnt signaling pathway, *SOX4* has attracted our attention because of its involvement in cell fusion in C2C12 (Jang et al., 2013). In addition, the RNA and protein levels of *SOX4* decreased with the knockdown of *H19* (Figures 4A,B). This result is consistent with transcriptome sequencing results, indicating that *SOX4* might be regulated by *H19*. Therefore, we chose *SOX4* for further exploration. The expression pattern of *SOX4* in PSCs showed that it was significantly upregulated during differentiation periods until differentiation for 24 h (Figure 4C), which also indicates that *SOX4* might play a role in the differentiation of PSCs.

The role of *SOX4* in cell differentiation was explored by designing and transfecting antisense oligonucleotides (ASO) into PSCs (Figure 4D). qPCR and Western blot results show that the expression levels of *MYOG*, *MYOD*, and *MYHC* genes decreased after *SOX4* was transfected with ASO (Figures 4E–G). We overexpressed the *SOX4* gene in PSCs and then induced their differentiation to confirm the knockdown results. As expected, *SOX4* overexpression significantly increased the expression of *MYOG* and *MYHC* at the mRNA and protein levels, but *MYOD* had no significant changes in the mRNA level, only in the protein level (Figures 4H–K). Interestingly, *H19* expression was not affected by *SOX4*, suggesting that *SOX4* was regulated by *H19* (Supplementary Figure S5A). Therefore, *SOX4* is regulated by *H19* and promotes the differentiation of PSCs.

### *H19* Regulated *SOX4* as a Molecular Sponge of *miR-140-5p*

**FIGURE 5:**
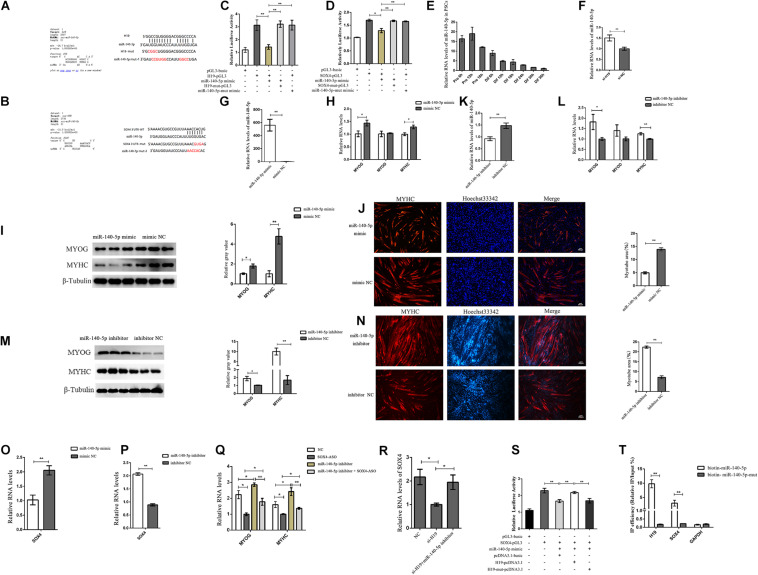
H19 regulated SOX4 as a molecular sponge of miR-140-5p. **(A)** Bioinformatics prediction of miR-140-5p as a target miRNA of H19 by RNAhybrid. **(B)** Bioinformatics predicted a miR-140-5p binding site in SOX4. **(C)** Luciferase reporter assay of H19-pGL3 and H19-mut-pGL3 in PSCs transfected with either miR-140-5p mimic or miR-140-5p-mut mimic. **(D)** Luciferase reporter assay of SOX4-pGL3 and SOX4-mut-pGL3 in PSCs transfected with either miR-140-5p mimic or miR-140-5p-mut mimic. **(E)** Real-time PCR analysis of miR-140-5p expression in PSCs during the period of proliferation and differentiation. There is a 6-h interval between each period. Mean values ± SEM, *n* = 3. **(F)** Real-time PCR analysis of miR-140-5p expression in PSCs transfected with H19 siRNA or negative control. **(G–I)** Overexpression of miR-140-5p inhibited the expression of MYOG and MYHC. **(J)** Representative photographs of MYHC immunofluorescence staining in PSCs differentiated for 24 h showing that miR-140-5p overexpression significantly decreased the MYHC expression level. **(K–M)** miR-140-5p inhibitor increased MYOG and MYHC level. **(N)** Representative photographs of MYHC immunofluorescence staining in PSCs differentiated for 24 h showing that miR-140-5p inhibitor significantly increased the MYHC expression level. **(O)** miR-140-5p overexpression decreased expression of the SOX4. **(P)** miR-140-5p inhibitor increased expression of the SOX4. **(Q)** Real-time PCR analysis of expression of MYOG and MYHC in PSCs after transfection with SOX4 ASO, miR-140-5p inhibitor, and a combination of the two. **(R)** Real-time PCR analysis of expression of SOX4 in PSCs after transfection with H19 siRNA or with H19 siRNA and miR-140-5p inhibitor. **(S)** Luciferase reporter assay of SOX4-pGL3 in PK cells transfected with miR-140-5p mimic, H19-pcDNA3.1, or H19-mut-pcDNA3.1. **(T)** Bintin-miR-140-5p pull-down was performed for PSCs transfected with biotin-miR-140-5p or mutated biotin-miR-140-5p, followed by real-time PCR to detect H19, SOX4 mRNA, and Gapdh mRNA levels. Mean values ± SEM, *n* = 3. The values are shown as means ± SD of three independent experiments.*P < 0.05. **P < 0.01.

*H19*, which is widely distributed in the cytoplasm, affects the expression of *SOX4*. Therefore, we speculated that *H19* might affect *SOX4* as a molecular sponge. Bioinformatics analysis revealed that *H19* had the putative recognition sequences for *miR-140-5p*, and *SOX4* had the potential binding site with *miR-140-5p* in its 3′ untranslated region (UTR) (Figures 5A,B). *miR-140-5p* mimic and mutant *miR-140-5p* mimic were synthesized to validate whether *H19* was indeed targeted by *miR-140-5p*. Luciferase reporters containing either a WT or mutant target site from *H19* were also constructed. *H19*-PGL3/*H19*-mut-PGL3 vectors and *miR-140-5p* mimic/mutant were cotransfected into PSCs. The luciferase activity analysis revealed that *miR-140-5p* mimic effectively inhibited the *H19*-PGL3 vectors’ luciferase activity, but the inhibition disappeared when the binding site or the *miR-140-5p* mimic was mutated (Figure 5C). These results strongly point to the mechanism that *H19* acts as a molecular decoy to abolish *miR-140-5p* repressing activity on its targets. In the following experiment, the WT *SOX4* 3′ and the mutant *SOX4* 3′ UTR binding sites were inserted into their respective luciferase reporter gene. Luciferase reporter assay revealed that *miR-140-5p* mimic, not mutant *miR-140-5p* mimic, could reduce the luciferase activity of *SOX4* 3′ UTR. However, the luciferase activity of the mutant *SOX4* 3′ UTR was not repressed by the *miR-140-5p* mimic (Figure 5D).

Next, we explored the function of *miR-140-5p*. *miR-140-5p* was significantly downregulated during differentiation periods as shown by its expression pattern in PSCs (Figure 5E). We detected the expression level of *miR-140-5p* after the knockdown of *H19* to understand the functional role of *H19* in *miR-140-5p* regulation. *H19* knockdown significantly upregulated the expression of *miR-140-5p*, but *H19* was not regulated by *miR-140-5p* (Figure 5F and Supplementary Figure S5B). In addition, the *miR-140-5p* mimic and inhibitor were transfected into PSCs to explore the function of *miR-140-5p*. The *miR-140-5p* mimic significantly decreased the expressions of *MYOG* and *MYHC* at the mRNA and protein levels and decreased the number of positive myotube formation (Figures 5G–J). An opposite result was identified in PSCs containing *miR-140-5p* inhibitor (Figures 5K–N). However, no significant difference was observed due to the low expression of *MYOD* after PSC differentiation. In addition, the *miR-140-5p* mimic reduced the expression level of *SOX4* (Figure 5O). Conversely, the *miR-140-5p* inhibitor increased *SOX4* expression (Figure 5P). These results imply that *miR-140-5p* inhibits the differentiation of PSCs by regulating *SOX4*.

Further confirmations were conducted to better explore the relationship between the three genes. As shown in Figure 5Q, the increased expressions of *MYOG* and *MYHC* induced by the *miR-140-5p* inhibitor were abolished by *SOX4* ASO. Simultaneously, the decreased *SOX4* expression induced by *H19* siRNA was rescued by *miR-140-5p* inhibitor (Figure 5R). Furthermore, the luciferase reporter assay shows that the luciferase activity of *SOX4*-pGL3 was increased upon the WT H19-pcDNA3.1 overexpression vector but not upon the *miR-140-5p* binding site mutated *H19*-mut-pcDNA3.1 vector (Figure 5S). The biotin-*miR-140-5p* pull-down assay also confirmed the interaction between *miR-140-5p* with *H19* and *SOX4* (Figure 5T). We concluded that *H19* might inhibit PSC differentiation by competitively binding *miR-140-5p* with *SOX4*.

### *H19* Directly Interacts With *DBN1*

**FIGURE 6:**
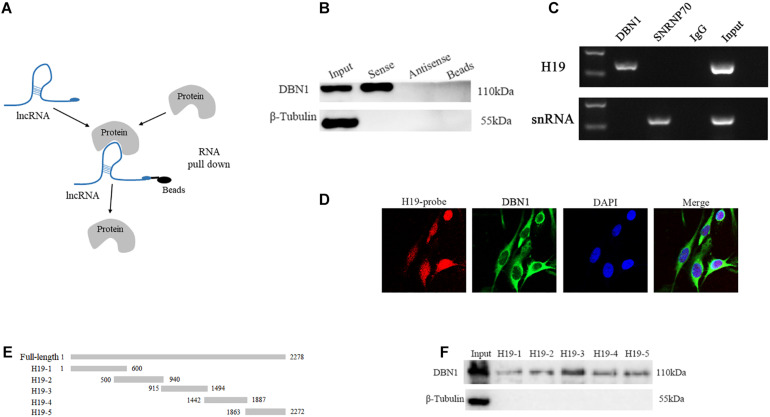
H19 interacts directly with DBN1. **(A)** A schematic representation of RNA pull-down assay. **(B,C)** Interaction between H19 and DBN1 as determined by RNA pull-down and RIP. **(D)** RNA FISH and immunofluorescence staining showed that H19 colocalized with DBN1 in PSCs cytoplasm. **(E,F)** The interaction of truncated H19 with DBN1 was determined by RNA pull-down.

We performed an RNA pull-down assay with biotinylated *H19* to identify the *H19* interacting proteins, followed by MS. The MS result is shown in Supplementary Table S4. The proteins that specifically bind to *H19* (including DBN1) are identified (Figure 6A). *DBN1* expression is induced during differentiation of primary and C2C12 myoblasts in a p38 MAPK-dependent manner (Mancini et al., 2011). Therefore, we selected DBN1 as the target protein. In the following experiments, the association of *H19* with DBN1 was validated by Western blot, and the results show that the target protein DBN1 is clearly detected in *H19* pull-down protein samples but not in the samples associated with antisense *H19* (Figure 6B). We performed RIP of DBN1 to validate this interaction between *H19* and DBN1. PCR results revealed that DBN1 pulled down more *H19* transcripts than IgG control in PSC cell lysates (Figure 6C). The results of *H19* RNA FISH and DBN1 immunofluorescence staining also show that the spatial localization of *H19* and DBN1 in PSCs is close (Figure 6D).

A series of truncated mutants of H19 were generated, and their binding capacity with DBN1 was tested to further map the binding domain (Figure 6E). Interestingly, all of the truncated mutants were capable of pulling down DBN1, but the RNA probes containing 915–1494 nts of the H19 gene pulled down more DBN1 protein (Figure 6F). These findings show that H19 directly interacts with DBN1.

### *DBN1* Affects PSC Differentiation

**FIGURE 7:**
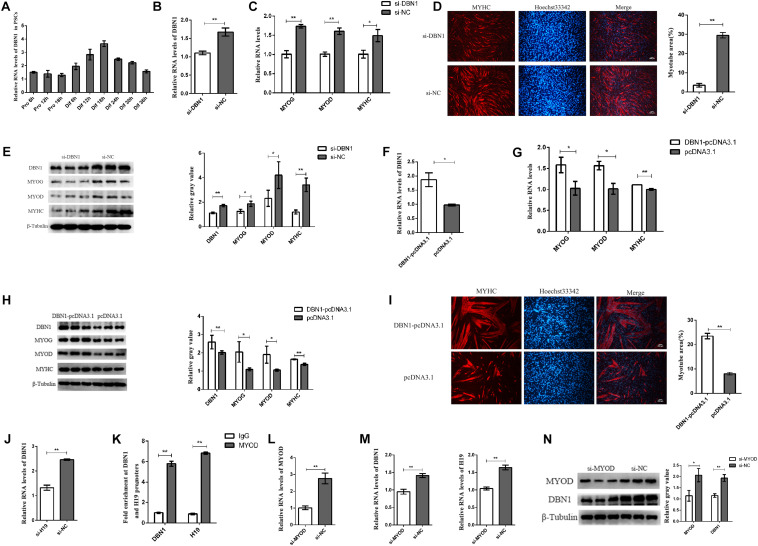
DBN1 affects the PSC differentiation. **(A)** Real-time PCR analysis of DBN1 expression in PSCs during the period of proliferation and differentiation. There is a 6-h interval between each period. SNRNP70 was used as positive control in RIP. Mean values ± SEM, *n* = 3. The values are shown as means ± SD of three independent experiments. **(B–E)** Knockdown of DBN1 inhibited the expression of MYOG, MYOD, and MYHC. **(F–I)** Overexpression of DBN1 promoted the expression of MYOG, MYOD, and MYHC. **(J)** Real-time PCR analysis of expression of DBN1 in PSCs after transfection with H19 siRNA. **(K)** ChIP-qPCR results revealed the enrichments of MYOD at the H19 and DBN1 promoters. **(L,M)** Knockdown of MYOD inhibited the expression of DBN1 and H19. **(N)** Knockdown of MYOD inhibited the protein level of DBN1. Mean values ± SEM, *n* = 3. The values are shown as means ± SD of three independent experiments. *P < 0.05, **P < 0.01.

The expression of *DBN1* in PSCs was detected to examine the role of *DBN1* in PSC differentiation. *DBN1* possessed a higher expression level in the differentiation period than in the proliferation period (Figure 7A). Then, *DBN1* was knocked down in PSCs by using siRNAs targeting *DBN1*, followed by induction of differentiation. qPCR and Western blot analyses revealed that *DBN1* knockdown significantly reduced the expressions of *MYOG*, *MYOD*, and *MYHC* (Figures 7B–E), whereas the overexpression of *DBN1* obtained the opposite results (Figures 7F–I), thereby indicating that *DBN1* is also necessary for PSC differentiation. Simultaneously, the downregulated expression of *H19* also decreased the expression of *DBN1*, but the expression of *H19* was not changed when *DBN1* was downregulated or upregulated (Figure 7J and Supplementary Figure S5C). *MYOD* is a transcription factor of *H19* and can affect the expression of *H19* (Borensztein et al., 2013), and *DBN1* is a target gene of *MYOD* (de la Serna et al., 2005; Forcales et al., 2012); therefore, we speculated whether *MYOD* also affects the expression of *DBN1* by performing ChIP-qPCR assays. The results show that *MYOD* could bind to the promoter of *DBN1* and *H19* (Figure 7K). Simultaneously, knocking down *MYOD* decreased the expression of *DBN1* and *H19* (Figures 7L–N). In summary, the knockdown of *MYOD* inhibited the expression of *H19* and *DBN1*, whereas the knockdown of *DBN1* or *H19* might also inhibit the expression of *MYOD.* We speculate that a feedback loop among the three genes might affect the differentiation of PSCs.

## Discussion

*H19* is one of the best-known imprinted genes discovered from several genetic screens in the liver, myoblasts, and embryonic stem cells (Pachnis et al., 1984; Poirier et al., 1991). Few articles discuss *H19* and muscle development, and most of these articles focus on mice and bovines. For example, *H19* performs a critical trans-regulatory function in skeletal muscle differentiation and regeneration by encoded microRNAs in mice (Dey et al., 2014). Xu et al. (2017) also demonstrate that *H19* can promote the differentiation of bovine skeletal muscle SCs by suppressing Sirt1/FoxO1. However, limited research has been conducted on PSC differentiation in pigs.

Myogenesis in pigs is a complex process that includes SC proliferation, differentiation, fusion, and specific muscle formation (He et al., 2017). This process requires precise regulation of genes with *H19* being one of the most important associated genes (Qin et al., 2017). We explored the function of *H19* in PSC differentiation by detecting first the expression pattern of *H19* in different tissues and different differentiation time points of PSCs. Then, we collected *H19* knockdown cells at various differentiation time points and determined the function of *H19* in regulating the differentiation of PSCs by high-throughput RNA-seq technology. *H19* knockdown suppressed the differentiation of PSCs in skeletal muscle development by suppressing three myogenic markers in RNA and protein levels.

In this study, a number of protein-coding genes were differentially expressed in PSCs at different time points of differentiation, especially in si36 versus NC36 and NC24 versus NC36. The identified DEGs are associated with normal physiological cell processes, such as metabolic pathways, endocytosis, and translation as well as muscle development pathways, including cell differentiation, positive regulation of cell proliferation, and Wnt signaling pathway. Many DEGs, such as *MSTN*, *TMEM8C*, *MYF6*, and *SOX4*, reportedly regulate myogenesis and were enriched in important pathways (Abmayr and Pavlath, 2012; Jang et al., 2013; Li et al., 2014; Retamales et al., 2015; Zhang et al., 2015; Millay et al., 2016). We also found that *FOXO1* was a DEG after *H19* knockdown. This indicates that *H19* might have a similar regulatory mechanism in pig and cattle, which deserves further investigation.

In recent years, many researchers have described lncRNAs containing miRNA binding sites that function as molecular sponges of miRNA, thereby modulating the derepression of miRNA targets and imposing an additional level of post-transcriptional regulation (Ebert and Sharp, 2010; Karreth and Pandolfi, 2013; Su et al., 2016). Among the abovementioned myogenesis-related genes, *SOX4* affects PSC differentiation and has a potential binding site for *miR-140-5p*, thus attracting our attention. *miR-140-5p* bound to the *SOX4* 3′-UTR region and affected its expression. We believed that *miR-140-5p* binding to *SOX4* 3′-UTR with incomplete complementarity inhibited protein synthesis but not mRNA degradation. The effect of *miR-140-5p* on *SOX4* mRNA level might be an indirect effect due to the influence of *miR-140-5p* on the differentiation process. *miR-140-5p* binds to *SOX4* 3′-UTR and inhibits the translation of *SOX4*, thereby inhibiting its protein expression. The effects of *miR-140-5p* on *SOX4* mRNA and protein levels might be realized through different mechanisms. *miR-140-5p* also has a potential binding site for *H19* as observed in bioinformatics analyses. Subsequent experimental results also show that *H19* acted mechanistically as a ceRNA to downregulate *miR-140-5p* and thereby upregulate the expression level of *SOX4*. Overexpression of *miR-140-5p* inhibits the differentiation of PSCs, whereas inhibition of *miR-140-5p* promotes the differentiation of PSCs. We conducted rescue experiments to verify the interaction of the three genes. The results of all these studies are consistent with our hypotheses that *H19* promotes the differentiation of PSCs by taking up *miR-140-5p* from *SOX4*. This is the first study to reveal *miR-140-5p*’s role in myogenesis.

Many lncRNAs perform their functions through their interaction with proteins (Huarte et al., 2010). For instance, lncRNA lrm regulates the expression of myogenic genes by directly binding to MEF2D, which, in turn, promotes the assembly of MyoD/MEF2D on the regulatory elements of target genes (Sui et al., 2019). In addition, *EPIC1* RNA promotes cell-cycle progression by interacting with MYC and enhancing its binding to target genes (Wang et al., 2018). Here, we focused our attention on *DBN1* because of its ability to promote myoblast differentiation (Mancini et al., 2011). We found by pull-down and RIP experiments that *H19* directly binds with DBN1 and promotes PSC differentiation synergistically. Moreover, all of the truncated mutants were capable of pulling down DBN1, but the RNA probes containing 915–1494 nts of *H19* gene pulled down more DBN1 protein, thereby suggesting that *H19* may be wrapped around the DBN1. *MYOD* is the main regulator during muscle differentiation (Endo, 2015; Zhang et al., 2018). *DBN1* as a target gene of *MYOD* like *H19* is also regulated by *MYOD* and further regulates the expression of *MYOD* (de la Serna et al., 2005; Borensztein et al., 2013), which further indicates the importance of *DBN1* in PSC differentiation. Considering the important roles of *H19* and *DBN1*, future efforts will be devoted to the detailed analysis of other diverse functional mechanisms through which *H19* and *DBN1* regulate PSC differentiation.

In reviewing the results of this study, some potential limitations should be considered. First, we only explored the function of *H19* from the aspect of knocking down *H19*. We have constructed an *H19* overexpression vector (*H19*-pcDNA3.1). However, due to the high expression of *H19* (much higher than *MYOG*, *MYOD*, and *MYHC*) in PSCs, *H19*-pcDNA3.1 could not increase the expression of *H19*, and PSC differentiation-related experiments were not conducted. We transfected the *H19* overexpression vector into PK15 cell lines and found that the overexpression effect was significant, thereby proving that the vector was successfully constructed (Supplementary Figure S5D). Hence, *H19* overexpression-related analysis experiments of luciferase activity were supplemented in PK15 cell lines. Second, our study revealed two cell differentiation pathways regulated by *H19*: one is to function as molecular sponges to inhibit *miR-140-5p*’s function and the other is to perform its function through their interaction with DBN1 proteins. However, the mechanisms of *H19* binding with DBN1 to regulate PSC differentiation have not been thoroughly studied. All these limitations deserve further investigation in the future.

**FIGURE 8:**
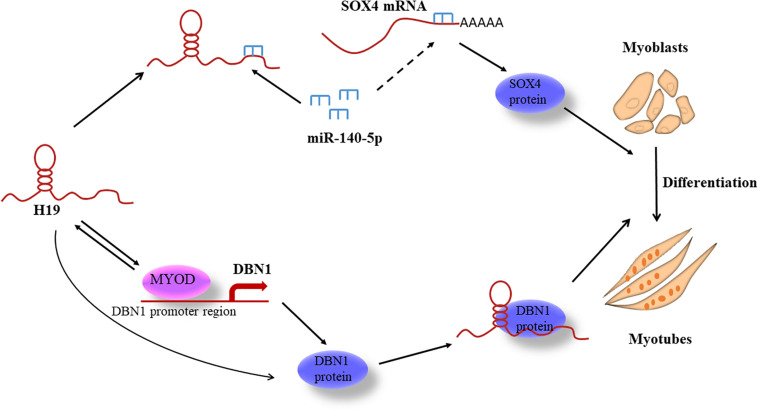
Model for H19-regulating PSC differentiation.

We present a molecular model to elucidate *H19*’s role in regulating PSC differentiation through two different pathways (Figure 8). This study is the first to identify and report *H19*-knockdown-mediated DEGs in porcine myogenesis and the first to uncover the mechanisms of *H19* in PSC differentiation that may provide some molecular basis for porcine myogenesis.

## Data Availability Statement

The RNA-seq was submitted to GEO. The accession number is GSE141648.

## Ethics Statement

All experimental protocols were approved by the Ethics Committee of Huazhong Agricultural University, Wuhan City, Hubei Province, China.

## Author Contributions

CL conceived and designed the experiments and explained the data. JL, TS, GS, and WL analyzed the main content of the data with the help of CL, CF, and CZ. JL and TS performed the experiment with the help of LC and GS. JL wrote the manuscript with the help of CL. All authors contributed to the article and approved the submitted version.

## Conflict of Interest

The authors declare that the research was conducted in the absence of any commercial or financial relationships that could be construed as a potential conflict of interest.
