# Supplementary material for: Long Non-coding RNA H19 Regulates Porcine Satellite Cell Differentiation Through miR-140-5p/SOX4 and DBN1
Source: Front Cell Dev Biol. 2020 Nov 25;8:518724. doi: 10.3389/fcell.2020.518724 (PMC7723966; doi:10.3389/fcell.2020.518724)
Supplement: Supplementary file 1 [file Table_1.DOCX]

**Table S1. Primers used for qPCR**

| **Gene** |  | | **Primer sequence(5’-3’)** | |
| --- | --- | --- | --- | --- |
| *H19* |  | F: CAGAACCCTCAAACGACAAGA | |  |
|  |  | R: TCCTTTGGCTCAACCTTCC | |  |
| *MYOG* |  | F: ATGAGACATCCCCCTACTTCTACCA | |  |
|  |  | R: GTCCCCAGCCCCTTATCTTCC | |  |
| *MYOD* |  | F: GGCTGCCCAAGGTGGAAATC | |  |
|  |  | R: TGCGTCTGAGTCACCGCTGTAG | |  |
| *MYHC* |  | F: GTTCAGAGAAAGGCATCCCAAA | |  |
|  |  | R: GAGAGTGACCGACACCACAAGTG | |  |
| *miR-140-5p* |  | Slp：GTCGTATCCAGTGCAGGGTCCGAGGTATTCGCACTGGATACGACCTACCA  F: AACACGCAGTGGTTTTACCC | |  |
|  |  | R: GTCGTATCCAGTGCAGGGT | |  |
| *SOX4* |  | F: CCTCAAGCACATGGCTGACTACCC | |  |
|  |  | R: CCACCGACCTTGTCACCCTTCTC | |  |
| *DBN1* |  | F: GATGAAGAAGTCAGAATCGGAGGTA | |  |
|  |  | R: CTGGTCGGTGGTTGAAGGGT | |  |
| *18S* |  | F: TCCCGACGTGACTGCTC | |  |
|  |  | R: GGTGACAGCGGGGTGG | |  |

**Table S2. Primers used for plasmid construction**

| **Primer name** | **Primer sequence(5’-3’)** |
| --- | --- |
| *SOX4* full-length sequence | F: CTAGCTAGCTTCACTTTAGCACGGTCTCCAGG |
|  | R: CCGGAATTCTTTCGCCATCAAGAACAACATCA |
| *DBN1* full-length sequence | F: TGAAGAACAAACACTTTCTCAGTCC |
|  | R: ACCCTCGAAGCCCTCCTCCT |
| *H19* full-length sequence  *H19* mut-length sequence  *miR-140-5p*、*H19* binding WT primer | F: AAGCAGGGTGAGGGAGGGGGGTG  R: TGAGTTTATTGATGAGTCCAGGGC  R1: AGCCGCGTGCTCCCTCCAG  F2: CTGGAGGGAGCACGCGGCT  F: GTGGAAAGAGCATCTCAAGCGAGTC |
|  | R: GCGCACAGTGCCACCAAGGA |
| *miR-140-5p*、*H19* binding mutant primer | R1: AGCCGCGTGCTCCCTCCAG |
|  | F2: CTGGAGGGAGCAGCCGCGT |
| *miR-140-5p*、*SOX4* binding WT primer | F: GGGGAGAATTAGCCAAACA |
|  | R: TTAGCGACAAGTAGAAAGG |
| *miR-140-5p*、*SOX4* binding mutant primer | R1: CCTCACGTTTAAACGGCACG |
|  | F2: AACGTGAGGATCTATCTAAATG |
| *H19* 1-600bp fragment | F: AAGCAGGGTGAGGGAGGGGGGTG |
|  | R: GCCGCCACTCTGCCGGGGGGTCAG |
| *H19* 500-940bp fragment | F: TTCCCTCCCCAGGCCTTGTT |
|  | R: ACTGCACCTTCTTTCCCATTCTCC |
| *H19* 915-1494bp fragment | F: CGGAGAATGGGAAAGAAGGTG |
|  | R: AGCCGAGAGTGTTCAGGAAGGC |
| *H19* 1442-1887bp fragment | F: TGACGGAGAGGGACAGACGTGA |
|  | R: GACAGAGGAAAAAAAGGAGGAAGGG |
| *H19* 1863-2272bp fragment | F: CCCTTCCTCCTTTTTTTCCTCTG |
|  | R: TGAGTTTATTGATGAGTCCAGGGC |

**Table S3. Primers used for RIP or ChIP**

| **Gene or Primer name** | **Primer sequence(5’-3’)** |
| --- | --- |
| *H19*-RIP primer | F: GAGTGGTCCTGATGTGGTAGTGG |
|  | R: GCCGTCGGCTGTAATTTGAT |
| *SnRNA* | F: GGGAGATACCATGATCACGAAGGT |
|  | R: CCACAAATTATGCAGTCGAGTTTCCC |
| *DBN*1-CHIP primer | F: GCTGGGAGGTTGGGATGGA |
|  | R: CCAAACTCTCACCTAGGGTGTTTCC |

**Table S4. The mass spectrum results of H19 pulldown**

| Description | Score | Unique Peptides | MW [kDa] |
| --- | --- | --- | --- |
| filamin-B isoform X7 [Sus scrofa] | 75.97 | 22 | 275.2 |
| drebrin [Sus scrofa] | 69.70 | 15 | 75.1 |
| vigilin [Sus scrofa] | 30.24 | 13 | 141.5 |
| heat shock cognate 71 kDa protein [Sus scrofa] | 29.91 | 13 | 70.8 |
| TAR DNA-binding protein 43 [Sus scrofa] | 11.125061 | 5 | 44.6 |
| insulin-like growth factor 2 mRNA-binding protein 2 [Sus scrofa] | 8.96 | 4 | 61.7 |
| tropomodulin-3 [Sus scrofa] | 8.89 | 4 | 39.6 |
| putative methyltransferase C9orf114 homolog [Sus scrofa] | 6.88 | 4 | 42.3 |
| RNA-binding protein FUS [Sus scrofa] | 6.30 | 3 | 52.4 |
| synaptopodin-2 isoform X2 [Sus scrofa] | 4.66 | 3 | 126.6 |
| proliferation-associated protein 2G4 [Sus scrofa] | 4.38 | 3 | 38.0 |
| myb-binding protein 1A [Sus scrofa] | 3.55 | 2 | 168.1 |
| myosin-13 [Sus scrofa] | 2.17 | 2 | 212.5 |
| fascin [Sus scrofa] | 1.77 | 2 | 54.6 |







Figure S1. Real-time PCR analysis of MYOD and MYHC expression in PSCs during the period of proliferation and differentiation, there's a 6h interval between each period. Mean values±s.e.m, n=3.


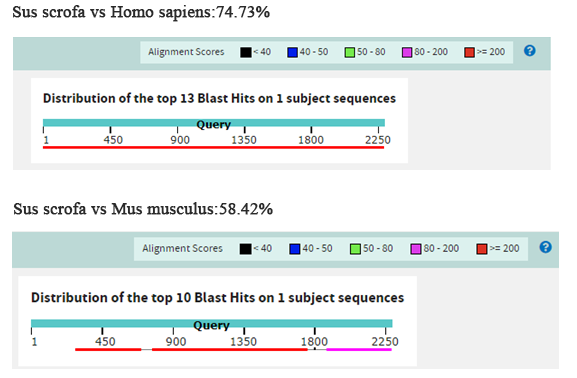


Figure S2. Conservation analysis of H19 gene among different species.


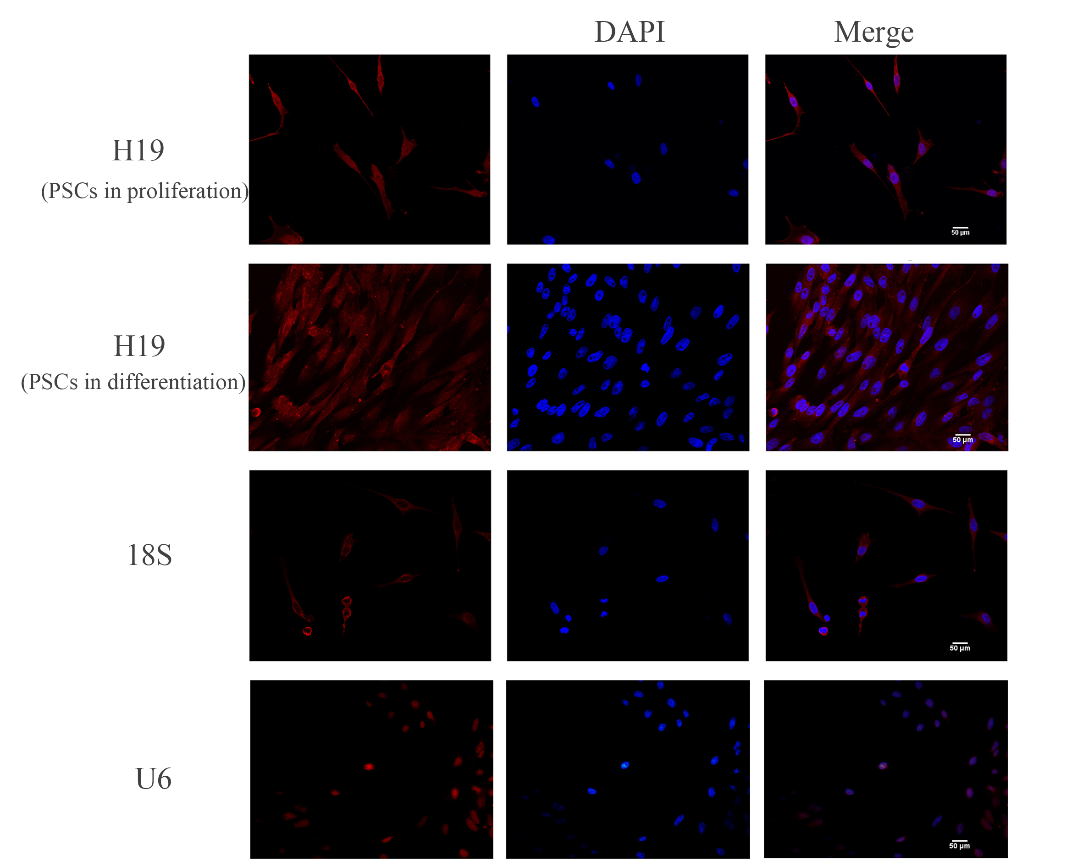


Figure S3. Confocal FISH images showing localization of H19 in PSCs. 18S, probe for 18S rRNA; U6, probe for U6 snRNA.


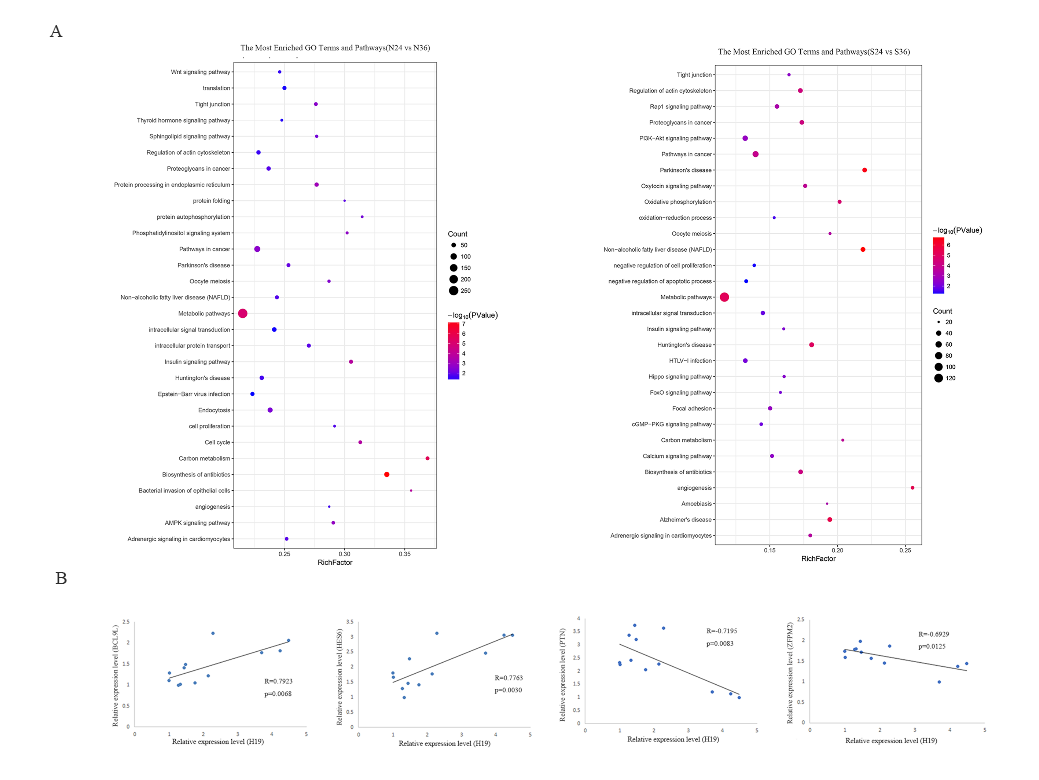


Figure S4. (A) The most enriched top 30 GO terms and KEGG pathways. Columns from bottom to top represent P values from small to large. (B) Linear regression of *H19* and selected DEGs expression. The R and P indicate the Pearson correlation coefficient and p value of each pair of *H19* and DEGs in twelve samples (three for the si24, three for the NC24, three for the si36, three for the NC36).


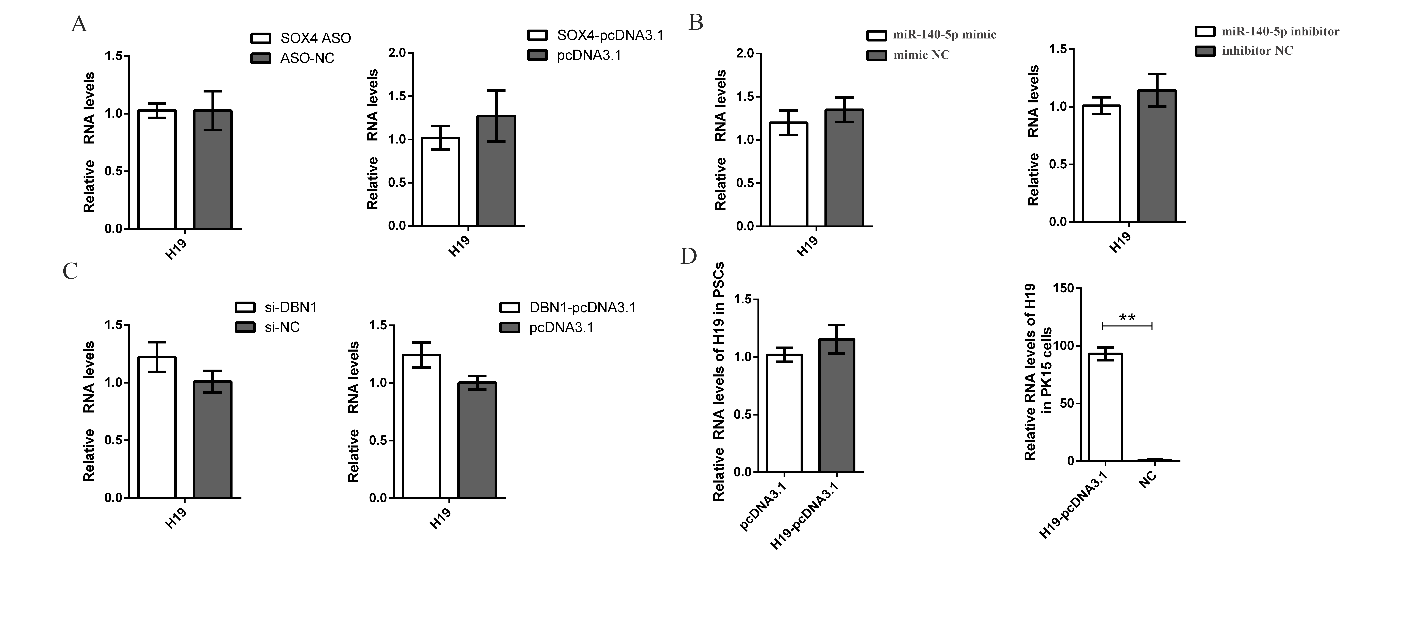


Figure S5. (A) The expression level of *H19* was not affected after knocking down or overexpressing *SOX4*. (B) The expression level of *H19* was not affected after overexpressing or knocking down *mir-140-5p*. (C) The expression level of *H19* was not affected after knocking down or overexpressing *DBN1*. (D) The overexpression levels of *H19* in different cell lines.

Sequence S1. Full length sequence obtained by Rapid-amplification of cDNA ends (RACE).

AAGCAGGGTGAGGGAGGGGGGTGGTGGGAGGGATGGGGGGGTTGGGGGAAACTGGGGAAGTGGGGGAGCCGAGGGGTAACCAGGGAAGCGGGGGTACCGGGGGCAACCAGGGGAAGATGGGGAAGCGGGGTGCAGGGGCGTTTGCGCGGGCCAAGGACCACCTTGGAAATCTGGAGCCTGGCAGGAGCGGCGCAGGGTTGAGGGGCTGGCTTGGGCAGGGCTGGCTGGCACCTGGGAGCCTGGCGGGGTTGAGGTCCGGGCTCCCAGGTGCCCTATAGGCAGGGCAACATCGGCATGGGGGGTGACAGGCCCGAGCTGGGGTGCGGAGGGAAGAGGGGGGAGCCAGGCATTCATCCCGGTCAATTTTGGTTTCAGGTCGTGGCGGCTGGTGGTCAGGGGGAGTTGGAGAGAGGTTCGCCCCGGGGCCTGGGGCAGCGGAGGTGTAGCTGGCAGCTGTGGGCAGGTGAGGACAGCCGTCTGCCGGGCCAGGTGAGTCCCCTTCCCTCCCCAGGCCTTGTTTCTCTGGCCTCCTGCATCCGGAGGTTCTGGGGAGCGAGGGCCGGCGAGGCGAAGCGGCTGACCCCCCGGCAGAGTGGCGGCGGACGACAGGCAAGGCGGGCAGAACAGGTGACACGTCTCAGGGGGAGCTGGGACCGGGCGGGGCTGGGGGGCCGGGGCCGTCCCAGGTGGAAAGAGCATCTCAAGCGAGTCTGGTGGGAGACGAGGCAGGGCTGCCAGCAGGGAGGAGACGCAACAGGCGGGGGGCATTCCAGGCCCGGGTCGGACAGGACCCGTCGGGGGTGTCAGGACAGTGGGGTCCCCAGCCGCCACTTCACCCACTGCAATTCATTTAGTAGCAGGTACAGGAGCGGCTCTGGCCGGGCCTCTTGAGGCCTGAGCTGGAGCCTCGAGGGCCGGAGAATGGGAAAGAAGGTGCAGTGTGCCAGACAGACGTCACCTGGAGGGAGCACGGCCGTGGGGACGGGCCCCAGAGAGATTTCGGCAGCAGGGAGGCTGCGCGGGCCCAGCCTGCGGACGTGCGTTCCCACGCAGCACTGCGGCCCAGGGGCTGGCGCGGCAGGGCCCCCGGTGTCCTTGGTGGCACTGTGCGCCCTCGCCGCTCGCCCCTGGGACTGGCACGGCAGACAGGACAGCACCCAGGGGAGTCAAGGGCACTGACGAGACCAGACTAGGCGAGGCGGGTGGGGTGGAATGGATGTGACCTCTGGGGGGAGGGAGGTGGGGACGCAGGCAGGGGCGAGGCGCCGGAGCCTGGCGGCGAGCGAGGCCAAGGCGGGCCTCTGCGGGTGACAACTGAGCACACATGGGTACCTTTGCGCTCGCACCGGAGACAGCCTCGTCTCCAGTGGGGGTGGACACACTGCCAGCACCACAGGCCGGACGCCAGGATGTGCTTGGAGGGACATGACACAGTCCGGTGTGACGGAGAGGGACAGACGTGACGCCGTCCGGCCTTCCTGAACACTCTCGGCTGGCCCCGCGGGGGAGCTGCCACACCCAGCGTCTGTTCCTTTGCCTTCCTGAAGGAGCACGTGCATGACTGCTGCTCTCTGGACCCCAGAACCCTCAAACGACAAGAGATGGTGCTACCCAGCTCATGCCTGGGCCTTGGACCCGGACTTCTTCAAGTCCTCCTAGCTCTGACTCAAGAATATGCTGCATTCTGGAGCCACTACACTACTTGACTCAGGAATCAGCTCTGGAAGGTTGAGCCAAAGGAACAGACGTCCCACACCACCGGACCAACGGCACCCGCGGGGTTCCCCACCCCCCGCCCGGCCACTCCACCTCGGCGGCCACCCCCTGCTGCGCCCTGGAGACACCACCAGCCTCCCTCTCTCCCCTTCCTCCTTTTTTTCCTCTGTCTTTTCTCTTCTCTTCTTTCCTCTCCTTTGCTCAGAAGACTCGGGGCATCCAGGACTCTGTGTCCCCGTCCTTCCTGAATTAATTTGCACTAAGTCGTTTGCACTGGTTTGGAGTCCTGGAACCAGCCCCGGGTCTCGGAGCGGGTGTGTGAGCTGCCGAGTGGCCTGGCCTCCTCGGCCCGCGCCCCCTCAGCACCTGCCATTGTCCATCTCTGTCTGGGGGTGACTGGGTGGGGGCCTGAGTGTGTGGGGCCCCGCCCTCCCCTCTCCTAGTCTGGAAGCTCCGACCACCGAGCAGACCTCAAACGCTGCACTGAGTGTCCATCTCGTCATGTGCCCCTCCTCGCCAGGGCCACCCCAGAGCCCTGGACTCATCAATAAACTCAGTTACCGGAAAAAAAAAAAAAAAAAAAAAAAAAAAAA
